# The RUDY study platform – a novel approach to patient driven research in rare musculoskeletal diseases

**DOI:** 10.1186/s13023-016-0528-6

**Published:** 2016-11-08

**Authors:** M. K. Javaid, L. Forestier-Zhang, L. Watts, A. Turner, C. Ponte, H. Teare, D. Gray, N. Gray, R. Popert, J. Hogg, J. Barrett, R. Pinedo-Villanueva, C. Cooper, R. Eastell, N. Bishop, R. Luqmani, P. Wordsworth, J. Kaye

**Affiliations:** 1 Oxford NIHR Musculoskeletal Biomedcial Research Unit, Nuffield Department of Orthopaedics, Rheumatology and Musculoskeletal Sciences, University of Oxford, Oxford, UK; 2 Nuffield Department of Population Health, University of Oxford, Oxford, UK; 3 MRC Lifecourse Epidemiology Unit, University of Southampton, Southampton, UK; 4 Academic Unit of Bone Metabolism, Metabolic Bone Centre, Northern General Hospital, Sheffield, UK; 5 Academic Unit of Child Health, University of Sheffield, Sheffield, UK; 6 The Botnar Research Centre, NIHR Oxford Musculoskeletal BRU, NDORMS, University of Oxford, Oxford, OX3 7HE UK

**Keywords:** Rare diseases, Database management systems, Dynamic consent, Patient reported outcome measures

## Abstract

**Background:**

Research into rare diseases is becoming more common, with recognition of the significant diagnostic and therapeutic care gaps. Registries are considered a key research methodology to address rare diseases. This report describes the structure of the Rare UK Diseases Study (RUDY) platform that aims to improve research processes and address many of the challenges of carrying out rare musculoskeletal disease research.

RUDY is an internet-based platform with online registration, initial verbal consent, online capture of patient reported outcome measures and events within a dynamic consent framework. The database structure, security and governance framework are described.

**Results:**

There have been 380 participants recruited into RUDY with completed questionnaire rates in excess of 50 %. There has been one withdrawal and two participants have amended their consent options.

**Conclusions:**

The strengths of RUDY include low burden for the clinical team, low research administration costs with high participant recruitment and ease of data collection and access. This platform has the potential to be used as the model for other rare diseases globally.

## Background

There are between five and eight thousand identified rare diseases [1–3]. Despite innovations in clinical management of more common diseases, up to a quarter of patients diagnosed with a rare disease can have a diagnostic delay of between 5–30 years and 95 % of rare diseases are still without specific treatments. The key concerns for patients and families are to have more information in terms of natural history and their long-term health [4]. Designing clinical trials for rare diseases requires a detailed understanding of the contemporary natural history of the disease to inform the selection of clinically important outcomes, inclusion/exclusion criteria, appropriate surrogate biomarkers and sample size calculations [5, 6]. Furthermore, transformative therapies for rare diseases are often costly and there is a growing need to establish the cost-effectiveness of such therapies in a real world setting, often within observational studies [7].

One of the main challenges to studying the epidemiology of rare diseases is in the ability to recruit and retain a representative sample of patients in longitudinal cohorts [7]. Patients are often located across wide geographical areas and institutions. This requires national, inter-institutional and potentially international collaborations to identify and contact patients. Individuals with rare diseases can also have significant mobility issues in addition to other health issues, which may limit their involvement in research studies that require frequent visits and/or travel over long distances. Finally, recruitment of patients attending secondary or tertiary care settings can result in only those with more severe phenotypes being studied, effectively excluding those with a milder phenotype. The value of each participant with a rare disease is high given the small pool of potential participants. This heightens the importance of designing studies that minimise participant attrition by maximising participant satisfaction and engagement.

## Methods

### Aim

To develop a novel web-based reporting study with a high level of participant engagement that improves understanding of aspects of rare diseases through research. The rationale of using a web-based approach was to address the issue of engaging patients over a wide geographical area and include those not attending secondary/tertiary care services. The aim of the high level of participant engagement is to ensure the study remains closely aligned to the needs of the participants to improve satisfaction, engagement and reduce attrition. The primary rare diseases of interest are osteogenesis imperfecta, fibrous dysplasia/McCune Albright syndrome, hypophosphatasia, melorheostosis, myeloma, pregnancy associated osteoporosis, X-linked hypophosphatemia, eosinophilic granulomatosis with polyangiitis, giant cell arteritis, granulomatosis with polyangiitis, polyarteritis nodosa and Takayasu arteritis. For patients who either do not recognize these definitions or have other rare disorders of the musculoskeletal system or blood vessels, they are able to enter their diagnosis in the ‘other diagnosis’ text box.

### Design

The RUDY study is currently designed as a five year prospective cohort study, funded by a partnership between the NIHR Rare Diseases Translational Research Collaboration and the Oxford NIHR Musculoskeletal Biomedical Research Unit, University of Oxford.

### Setting

**Fig. 1:**
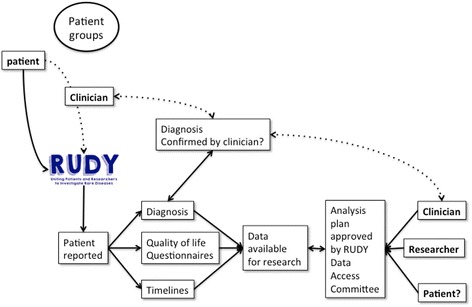
Schematic of interactions between patient groups, patients, participants, clinicians and academics within the RUDY Study

RUDY is a UK based national network of doctors, researchers, patient groups, patients and families working collaboratively to improve understanding of rare diseases and enable the development of new tests and treatments (Fig. 1).

### Development

Early in the design phase, the key patient groups in each disease area were identified and patient representatives were invited to join a Skype call to discuss potential design aims. The patient groups identified three key concerns: a) the lack of a comprehensive record of their care and treatment with substantial issues when transferring from paediatric to adult care, between specialists and between hospitals; b) the lack of reliable information on the Internet; c) significant regional variations in care despite similar disease severity. It was also clear that there was a lack of contemporary quality of life and other patient reported outcomes in many rare diseases, coupled with a lack of validated disease specific tools. From the clinical staff perspective, it was clear there was a need to minimize clinician/research nurse burden in terms of recruitment and entering data if there was going to be high uptake to RUDY, this would also cut down on costs for recruitment and data entry. This led to the decision to base the study on-line and implement a generic suite of patient reported assessment tools covering quality of life as well as other aspects such as pain, fatigue, sleep, anxiety and depression with minimal burden on clinicians.

Governance structures: The Study Management Group (SMG) manages the day-to-day running of RUDY. The SMG meets weekly to: report on the development of RUDY, including new features; respond to comments from the patient forum and participants and deliver the immediate and short terms goal of RUDY. The SMG is made up of the principal investigator, project director, database developer, data manager and ethicist. We had initially included patients in the SMG but the attendance to the weekly meeting was low, reflecting a high burden, and so the patient engagement is principally performed through the patient forum. Transparency in RUDY was considered important to support engagement with stakeholders. Three governance bodies were created to enable the smooth running of the project and to ensure that the collection, management, use and access to patient’s data was conducted in a transparent and accountable manner. These bodies are: the Data Outcomes Committee which deals with issues related to the database content, the Data Access Committee which reviews and approves submitted analysis plans and other data access requests and the Policy committee generates the terms of publication of findings using the RUDY study data. Each of these committees has a Chairperson, representation from the SMG and Patient Forum and is ultimately responsible to the SMG. Finally, an external advisory board has been developed to advise on the strategic direction of RUDY.

### Eligibility

Eligible participants include patients with a rare disease, blood relatives and partners of patients with a rare disease. Individuals aged 0–100 years are eligible and a rare disease is defined by a prevalence rate of less than 1 in 2,000 [8]. For those aged below 18 years the permission of a parent or guardian is required as well as assent of the child.

### Recruitment

Registration is done by the patient or their carers online. The involvement of clinical staff is minimal with the opportunity for clinician help if needed (Fig. 1). The web address (https://research.ndorms.ox.ac.uk/rudy) has been circulated to the major patient groups for rare diseases of the musculoskeletal system and blood vessels through presentations to patient groups as well as through Google, Facebook and Twitter. Recruitment has also come via word of mouth, with clinicians or patients recommending the site to others.

The patient information sheets, guide to registration, consent forms and assent forms for adults and children are available to download as PDFs in the library section of the website. Interested parties are invited to register online in a three-stage process that includes patient identifier and contact details, diagnosis and finally a calendar function for when they want to be contacted by telephone. The diagnosis is selected from a pre-defined list and should their diagnosis not be present, it can be directly entered into the ‘other diagnosis’ box.

The registration form is sent to the research administration team and an appointment time is confirmed with the participants by email. This form has a high security with 256-bit encryption. The patient is contacted by telephone and a trained researcher follows a script to explain the study, ask for any questions and to ask for the patient’s consent. Participants who give their verbal consent are sent, by email or post, a consent form to complete and return to the RUDY Study group. The diagnosis of each participant is verified through contact with the patient’s current clinician either in secondary or primary care. This is usually in the form of a copy of an appropriate outpatient clinical letter that includes the participants name, address and diagnosis. Consent is dynamic, allowing patients to alter their consent options online in terms of access to health records, use of samples and data and contact for other studies and updates.

### Participant engagement and dynamic consent

From the outset, the aim of RUDY was to have a high level of participant engagement and this is delivered in part through dynamic consent [9]. Informed consent is a fundamental ethical requirement in clinical research involving human participants. In face-to-face, paper –based consent models, discussion between the research team and participant for consent is a process that occurs at the beginning of a study. It is then assumed that the participants retain this record and do not change their consent options unless they decide to withdraw from the study. The dynamic consent model is an on-going two-way dialogue between participants and researchers. Participants are able to view their consent options on line at any time within their secure web-space. Participants can decide to change parts of their consent (e.g. use of surplus tissue for research). Should this happen any data/sample that could be affected is not destroyed but is no longer made available for research as described in the participant information sheet. Data and samples that have been already anonymized are not affected. Other consent options include contacting participants about clinical studies, collection of surplus tissue and linkage to other research databases.

Integral to participant retention is that participants value being a part of the study. As part of this two-way process, researchers post lay summaries of abstracts and papers on the profile page of every participant that has contributed data and/or samples. Participants can contribute to the on-going development of the study through continuous two-way communication with the research team.

The aim is to improve participant retention but also to improve the efficiency of the study design, implementation and subsequent development. For those participants who wish to play a greater role in the RUDY study, they can join the Patient Forum that functions in three ways: a) through six weekly Skype calls in the evening that last up to 60 min. These are loosely structured around feedback on current use of RUDY, upcoming RUDY website updates, queries from SMG, and prioritization of future work; b) on-going access to a secure online RUDY test site where new or updated website features are available to be tested by the forum before they are implemented in the main RUDY website; c) discussion of new research areas. For example, following guidance from the Patient Forum, the study was restructured into a main web-based study with, currently, four sub-studies including clinic visits for assessment of physical function, blood and urine tests, bone density test and skin biopsies. The Patient Forum has also requested: inclusion of questions that measure fatigue, a module where patients can enter information about how their rare disease has affected education, employment and social activities, a diary of their health care usage, presence on Facebook and Twitter and the inclusion of family members and partners. All of these changes have required a substantial amendment to the original research ethics committee approval, which has been given greater weight because the Patient Forum has requested these changes.

The annual stakeholder meeting provides the opportunity for interested parties to be kept up to date and maintain their involvement in the project. This meeting includes members of the Patient Forum, clinicians, researchers and the SMG.

### Data collection

**Fig. 2:**
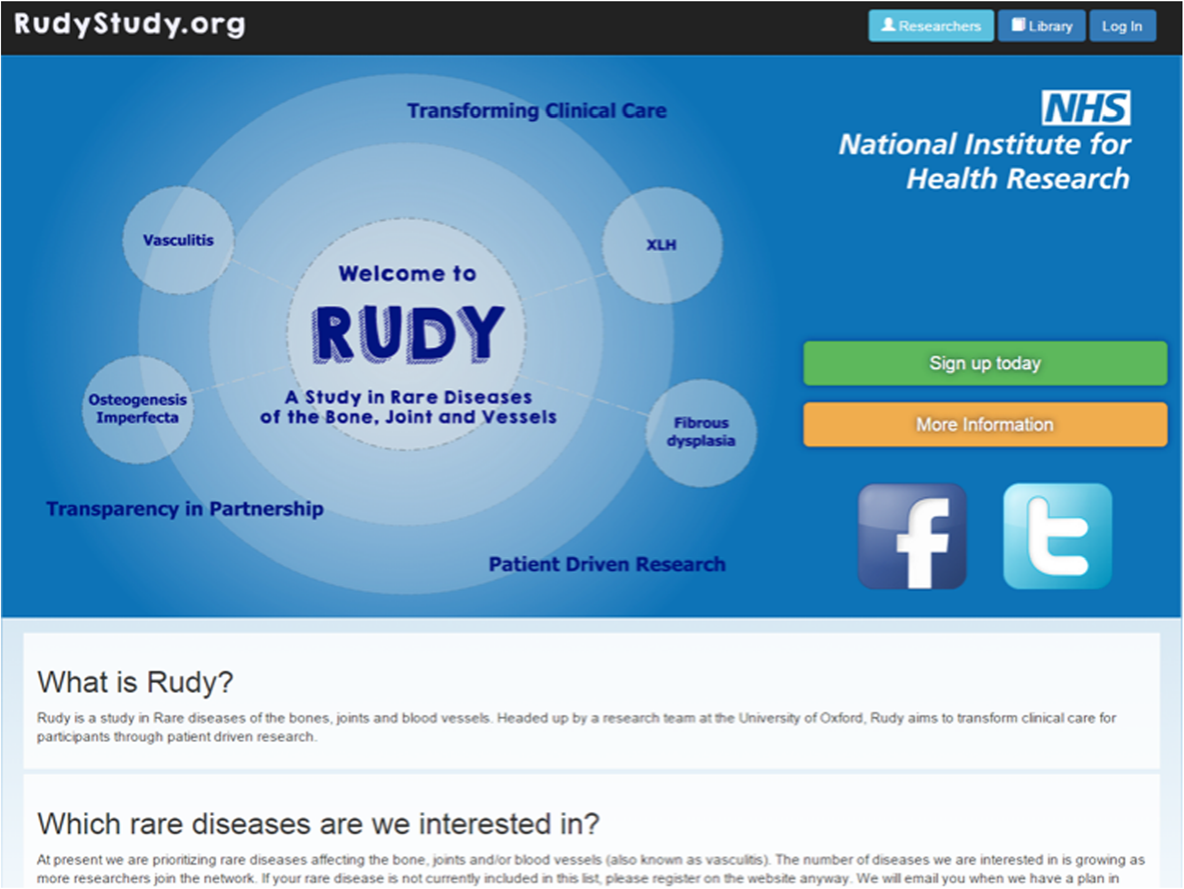
Homepage of the RUDYstudy.org

Once consent is gained, participants are granted full access to a secure personalised homepage on the study website (Fig. 2). To allow for future linkage to other national databases, additional demographic information including postal address and items needed for the Global Unique Identifier [10] are collected in addition to listing their current secondary and primary care clinicians. Participants are then directed to their landing page that has three broad sections: “To do”, “Profile” and “Timeline”. On the “To do” page is a list of questionnaires to be completed online that are colour-coded depending on their completion, with blue indicating new questionnaires, orange for questionnaires that have been started but are incomplete, and green for those that have been completed.

Validated patient reported outcome questionnaires used include, for adults, the EQ5D-5 L [11], SF-36 [12], Nottingham Activities of Daily Living score activity [13], Pittsburgh Sleep Quality Index [14], McGill pain [15], PainDetect questionnaires [16], Hospital Anxiety and Depression scale [17], Functional Assessment of Chronic Illness Therapy- Fatigue (FACIT-i); for children: the PedsQL, Childhood Health Assessment Questionnaire [18] and the Wong-Baker Faces questionnaires [19]. For adults with vasculitis: the Rasch-built Overall Disability Scale (R-ODS) for inflammatory neuropathy [20] and vasculitis specific Patient reported outcome [21]. Each questionnaire has been checked where appropriate by the respective licensing authority to confirm the web version is an acceptable representation of the paper form.

To record significant clinical events, participants are offered a timeline to add events interactively. Participants record the time in calendar day, month and/or year or a range of years. At present this is focused on fractures and includes the level of trauma and the site of bones broken. There is an optional facility to record aspects of management, including hospital site, length of hospital stay, number of operations, outpatient attendance, medications, physical therapy, recovery, use of mobility aids and complications such as infection, delayed healing and need for repeat surgery. These results are then presented on a timeline for participants to view as a diary format. The aim of the diary format is two-fold: firstly, to satisfy a need from participants to be able to record their clinical events in a secure and easily accessible format; and secondly, to inform understanding of the natural history of rare diseases.

### Clinical Research opportunities

In addition to recruiting participants to the study and inviting them to complete questionnaires, within the RUDY ethics there are embedded sub-studies for clinical assessment, blood and urine testing, skin biopsies for generation of cell lines as well as body composition assessment using dual energy X-ray Absorptiometry. In addition, the RUDY study cohort represents a registry of phenotyped patients who are asked whether they are happy to be recontacted for other research studies as, and when, approved by the Rudy Data Access Committee. This allows other research groups for studies and trials to approach RUDY for contacting potentially eligible participants.

### RUDY programming and security

RUDY is built with Laravel PHP open source framework and has a modular design by a team of professional software and web-developers mapping to the Rare Disease Registry check list [22]. This includes clear project management, team based development, well-structured commented source code and development guide, version control and issue tracking of software and documentation. The database information security complies with UK NHS information toolkit v12 and all identifiable data is 256 bit encrypted. Participant-identifiable data is only accessible to the participant and the specific administrators who need access to identifiable data to arrange appointments, respond to queries and update confirmation of diagnosis from clinicians. RUDY also includes specific code to support future software developments and linkage of RUDY information with other research databases and information such as external tables of hospitals or medications.

Researchers can only access non-identifiable data and this can be exported in a comma separated-values (CSV) format. Every user login to the RUDY database is recorded in addition to software updates with a record for what data was added, updated or deleted, by whom and at what date and time. Access to individual records is also recorded.

### Social media involvement

Many rare disease groups have private Facebook pages and discussion websites for patients. Following advice from the Patient Forum, it was evident that presence on social media would be beneficial for RUDY. It was also clear that the RUDY Facebook page should not overlap with existing closed Facebook pages and instead aim to a) increase the profile of RUDY externally, b) improve recruitment c) provide another method to offer updates on RUDY. We submitted and gained ethical approval to create a separate Facebook page (RUDYstudy) and twitter account (@Rudystudy) for the RUDY study. These are linked to the RUDY study website as well as the social media pages of our charity and patient group partners such as the Brittle Bone Society and Fibrous Dysplasia Support Society UK. Posts and tweets include updates on the number of patients recruited, new online study events and website features, grants awarded and results of research. To date the Facebook page has had 207 likes and the Twitter account 46 Followers.

## Results

Initial approval for the study was gained from the South Central Research Ethics committee after review (LREC 14/SC/0126) in April 2014. The RUDY platform was launched in June 2014 and by March 2016, the study had recruited 380 participants, comprising 336 adults and 46 children with the diagnoses shown in Table 1. Approximately 20 % of requests for clinical confirmation using a clinic letter have been returned.

**Table 1:** Recruitment of participants by diagnosis up to March 2016

Diagnosis	Paediatric	Adults
Osteogenesis imperfecta Type I	9	25
Osteogenesis imperfecta Type III	1	13
Osteogenesis imperfecta Type IV	1	10
Osteogenesis imperfecta Type V/VI/VII	0	0
Osteogenesis imperfecta Type unknown	2	23
Fibrous Dysplasia	6	59
X-Linked Hypophosphataemia	3	30
Hypophosphatasia	0	7
Melorheostosis	0	2
Eosinophilic granulomatosis with polyangiitis	0	23
Granulomatosis with polyangiitis	0	50
Microscopic polyangiitis	0	14
Polyarteritis nodosa	0	3
Takayasu arteritis	0	6
Other rare diagnoses	24	69

Table 2 shows the proportion of adult questionnaires completed for all diseases at baseline and 6 months follow-up. The completion rate of questionnaires was higher at 6 months (70.1 %) than baseline (56.8 %). The completion rate was over 60 % at baseline and 75 % at 6 months for 5/5 for the vasculitides; of the rare bone diseases, 3/5 diseases had greater than 60 % completion at baseline and 0/5 over 75 % at 6 months. We then compared the completion of the two quality of life measures at baseline and follow-up by diagnosis for the most common rare diseases (Table 3). The completion rates at baseline varied from 54–100 % by disease. Of the questionnaires, the EQ-5D5L tended to be slightly better completed than the SF36 at baseline with no consistent difference at 6 months.

**Table 2:** Baseline and 6 months completion of patient reported outcomes questionnaires

Domain	Questionnaire	Baseline (%)	6 months (%)
Quality of Life	EQ-5D 5 L	215/370 (58)	124/181 (69)
	SF-36	207/370 (56)	128/181 (71)
Pain	McGill Long	210/370 (57)	123/181 (68)
	Pain detect	200/370 (54)	129/181 (72)
Activities of Daily Living	Nottingham ADL	219/370 (59)	124/181 (69)
Sleep	Pittsburgh Sleep Quality Index	209/370 (57)	124/181 (69)
Fatigue^a^	FACIT-Fatigue	93/212 (44)	135/181 (75)
Depression^a^	Hospital Anxiety and Depression Scale	94/212 (44)	123/181 (68)

Participants were scheduled for the 6 months questionnaires after completing at least one questionnaire at baseline

^a^These two questionnaires were introduced after a substantial amendment and those participants who had already completed a baseline questionnaire had these questionnaires scheduled with their next assessment at 6 months

**Table 3:** Completion of Adult questionnaires at baseline and 6 months by diagnosis

Diagnosis	Baseline		6 months	
	EQ5D	SF36	EQ5D	SF36
Osteogenesis imperfecta	40/71 (56 %)	38/71 (54 %)	21/36 (58 %)	22/36 (61 %)
Fibrous Dysplasia	40/59 (68 %)	38/59 (64 %)	19/28 (68 %)	17/28 (61 %)
X-LH	24/30 (80 %)	22/30 (69 %)	15/22 (68 %)	
Hypophosphatasia	3/7 (43 %)		1/2 (50 %)	
Melorheostosis	2/2 (100 %)		0/0	
Eosinophilic granulomatosis with polyangiitis	14/23 (61 %)	13/23 (56 %)	9/12 (75 %)	
Granulomatosis with polyangiitis	33/50 (65 %)		25/31 (81 %)	27/31 (87 %)
Microscopic polyangiitis	9/14 (69 %)		4/7 (57 %)	6/7 (86 %)
Polyarteritis nodosa	2/3 (67 %)		1/2 (50 %)	2/2 (100 %)
Takayasu arteritis	5/6 (83 %)		4/5 (80 %)	

Number of completed per number of submitted questionnaires by disease group shown, with percentages in parenthesis

To date, one participant has withdrawn and two participants have changed their consent options both to opt in to either family tree linkage or linking of their data to other research studies.

## Discussion

Rare disease registries are considered to be a key component of research improving outcomes for patients. There is an increase in the profile of research in rare diseases [23] supported by changes in health policy in a number of countries [24]. A large number of rare disease registries are currently being used globally [25, 26] and this has led to the development of common elements for databases including those within the EPIRARE [10] and GRDR [27] programmes. However, online interaction registries are still in their infancy and there are a number of challenges to be addressed before they become a common research methodology.

There was little difference between completion rates between questionnaires. The higher completion rate at 6 months vs. baseline suggests that once patients had started to complete questionnaires at baseline, they were motivated to continue completing them at their next follow-up. When comparing the shorter EQ5D-5 L with the longer SF36, completion rates were only slightly higher for the EQ-5D at baseline with no consistent difference at follow-up. Further work is needed to determine the benefit of measuring both. Between the diseases, there was variation in completion rates, with highest proportions in patients with vasculitis compared with rare bone diseases. Participants with vasculitis are more likely to be recently diagnosed and unwell and this may explain the higher reporting rate. More work is required to explore reasons why participants did not complete their assessments and assess whether clearer guidance is required to improve completion rates. For example, when these findings were discussed with our Patient Forum, it became clear that participants without pain, may not realise they still need to complete the pain assessments or be motivated to do so.

The RUDY database brings together several unique features distinct from many other registries such as online registration, dynamic consent and participant entry of data in addition to significant involvement in the decisions made about the creation, design and management of the platform. This partnership with patients and patient groups has been aimed at ensuring the optimal experience for people in taking part in RUDY. This approach has the additional benefit of minimizing the time clinical and researcher staff spend on recruitment and data entry, improving recruitment and retention. Feedback from patients has shown that they find the dynamic record keeping and the ability to print off their data for personal use particularly valuable, which supports the findings from other internet-based registries [28]. We propose that if patients are aware of exactly what they are contributing to, and are updated with how it is progressing the evidence base for their disease, they will see themselves as partners in the project, and thus may feel more empowered

Current research platforms, such as RD-Connect, focus on developing a combined repository by cross-linking data across clinical phenotypic and genomic data and making this available for researchers [29]. The RUDY platform informs the clinical phenotype with patient entry of phenotyping data within a dynamic consent framework. It is appropriate for patients with rare diseases who live in geographically diverse areas because it allows frequent interaction without the significant burden of travelling time and costs. Compared with more traditional study models that require face-to-face interactions between participants and researchers, this model enables greater numbers to be recruited and it could be used for participants based in other countries. Such an international platform would deliver common data collection with translation as appropriate with the required linkage to national ethical and other regulatory approvals. Use of similar web based portals with dynamic consent may be applicable to research in other rare conditions and also be effective in more common diseases.

There are a number of challenges to carrying out an Internet based study. We were concerned that a number of potential participants would not have access to the Internet (or would not wish to use the Internet for this purpose) and that this would restrict recruitment to those with Internet knowledge and access. Our experience has been that most patients have managed to access their online page. The protocol provides that if Internet access is not possible then paper-based forms can be posted to individuals. To date, no participant has needed paper-based support, because family members have been able to provide Internet access. We recognize that this may be specific to countries with reasonable Internet access.

Two of the major concerns of patients were about the quality of information on the Internet and variable care between hospitals. Addressing these issues is challenging, especially for the undiagnosed or newly diagnosed patient. Providing evidence and consensus for care pathways, both nationally and internationally, is one potential solution and is actively being addressed for fibrous dysplasia/McCune Albright Syndrome. Applying a fixed age threshold for using participant consent vs. parent/guardian consent with participant assent is challenging. A number of authors have challenged the acceptability and quality of childhood assent in the absence of a check of the child’s maturity. The age threshold of 18 years was a pragmatic decision.

Another difference between paper-based systems and online systems is establishing a mean of confirming the identity of participants registering on-line as well as confirming the diagnosis. This is currently being performed using a confirmation of diagnosis from their current clinician with only a minority returning the information and accounts for the large number of “Other” diagnoses. Requesting copies of the clinical records from participants may be a solution. The completion of the baseline questionnaire rate needs further work to encourage higher completion rates. While we recognize that RUDY has substantial participant influence, the lack of a set of criteria and standards means that this cannot be quantitatively measured and reported. Further work is needed to identify the metrics for patient-centric platforms to enable benchmarking and standard setting. Finally, paediatric recruitment is lower than for adults and future work is needed to understand the reasons for this and to guide future development of RUDY.

## Conclusions

To meet the needs of patients, clinicians and researchers, we describe an innovative patient-driven web-based platform with explicit and dynamic consent of participants, online data assessment and engagement that could be used as a model to follow in the development of other rare disease databases.

## Abbreviations

RUDY: Rare UK diseases study
SMG: Study management group

## References

[1] Schieppati A, Henter JI, Daina E, Aperia A (2008). Why rare diseases are an important medical and social issue. Lancet.

[2] Griggs RC, Batshaw M, Dunkle M, Gopal-Srivastava R, Kaye E, Krischer J (2009). Clinical research for rare disease: opportunities, challenges, and solutions. Mol Genet Metab.

[3] Wastfelt M, Fadeel B, Henter JI (2006). A journey of hope: lessons learned from studies on rare diseases and orphan drugs. J Intern Med.

[4] Cavero-Carbonell C, Gras-Colomer E, Guaita-Calatrava R, Lopez-Briones C, Amoros R, Abaitua I, et al. Consensus on the criteria needed for creating a rare-disease patient registry. A Delphi study. J Public Health (Oxf). 2015;38(2):e178–e186. 10.1093/pubmed/fdv099 26294444

[5] Augustine EF, Adams HR, Mink JW (2013). Clinical trials in rare disease: challenges and opportunities. J Child Neurol.

[6] Clarke JT, Coyle D, Evans G, Martin J, Winquist E (2014). Toward a functional definition of a "rare disease" for regulatory authorities and funding agencies. Value Health.

[7] Potter BK, Khangura SD, Tingley K, Chakraborty P, Little J. Translating rare-disease therapies into improved care for patients and families: what are the right outcomes, designs, and engagement approaches in health-systems research? Genet Med. 2016;18(2):117–23. 10.1038/gim.2015.42 25856667

[8] EURODIS. EURODIS: the Voice of Rare Disease Patients in Europe 2015 [Available from: http://www.eurordis.org. Accessed 03 Apr 2016.

[9] Kaye J, Whitley EA, Lund D, Morrison M, Teare H, Melham K (2015). Dynamic consent: a patient interface for twenty-first century research networks. Eur J Hum Genet.

[10] Taruscio D, Mollo E, Gainotti S, Posada de la Paz M, Bianchi F, Vittozzi L (2014). The EPIRARE proposal of a set of indicators and common data elements for the European platform for rare disease registration. Arch Public Health.

[11] Rabin R, de Charro F (2001). EQ-5D: a measure of health status from the EuroQol Group. Ann Med.

[12] Jenkinson C, Coulter A, Wright L (1993). Short form 36 (SF36) health survey questionnaire: normative data for adults of working age. BMJ.

[13] Lincoln NB, Gladman JR (1992). The Extended Activities of Daily Living scale: a further validation. Disabil Rehabil.

[14] Buysse DJ, Reynolds CF, Monk TH, Berman SR, Kupfer DJ (1989). The Pittsburgh Sleep Quality Index: a new instrument for psychiatric practice and research. Psychiatry Res.

[15] Melzack R (1975). The McGill Pain Questionnaire: major properties and scoring methods. Pain.

[16] Freynhagen R, Baron R, Gockel U, Tolle TR (2006). painDETECT: a new screening questionnaire to identify neuropathic components in patients with back pain. Curr Med Res Opin.

[17] Zigmond AS, Snaith RP (1983). The hospital anxiety and depression scale. Acta Psychiatr Scand.

[18] Varni JW, Seid M, Smith Knight T, Burwinkle T, Brown J, Szer IS (2002). The PedsQL in pediatric rheumatology: reliability, validity, and responsiveness of the Pediatric Quality of Life Inventory Generic Core Scales and Rheumatology Module. Arthritis Rheum.

[19] Wong DL, Baker CM (1988). Pain in children: comparison of assessment scales. Oklahoma Nurse.

[20] van Nes SI, Vanhoutte EK, van Doorn PA, Hermans M, Bakkers M, Kuitwaard K (2011). Rasch-built Overall Disability Scale (R-ODS) for immune-mediated peripheral neuropathies. Neurology.

[21] Robson JC, Milman N, Tomasson G, Dawson J, Cronholm PF, Kellom K (2015). Exploration, Development, and Validation of Patient-reported Outcomes in Antineutrophil Cytoplasmic Antibody-associated Vasculitis Using the OMERACT Process. J Rheumatol.

[22] Bellgard M, Beroud C, Parkinson K, Harris T, Ayme S, Baynam G (2013). Dispelling myths about rare disease registry system development. Source Code Biol Med.

[23] Baxter K, Terry SF (2011). International Rare Disease Research Consortium commits to aggressive goals. Genet Test Mol biomark.

[24] Rodwell C, Ayme S (2015). Rare disease policies to improve care for patients in Europe. Biochim Biophys Acta.

[25] Santoro M, Coi A, Lipucci Di Paola M, Bianucci AM, Gainotti S, Mollo E (2015). Rare disease registries classification and characterization: a data mining approach. Public Health Genomics.

[26] Taruscio D, Gainotti S, Mollo E, Vittozzi L, Bianchi F, Ensini M (2013). The current situation and needs of rare disease registries in Europe. Public Health Genomics.

[27] Rubinstein YR, Groft SC, Bartek R, Brown K, Christensen RA, Collier E (2010). Creating a global rare disease patient registry linked to a rare diseases biorepository database: Rare Disease-HUB (RD-HUB). Contemp Clin Trials.

[28] Johnson KJ, Mueller NL, Williams K, Gutmann DH (2014). Evaluation of participant recruitment methods to a rare disease online registry. Am J Med Genet A.

[29] Thompson R, Johnston L, Taruscio D, Monaco L, Beroud C, Gut IG (2014). RD-Connect: an integrated platform connecting databases, registries, biobanks and clinical bioinformatics for rare disease research. J Gen Intern Med.

